# Stem rust in Western Siberia – race composition and effective resistance genes

**DOI:** 10.18699/VJ20.608

**Published:** 2020-03

**Authors:** V.P. Shamanin, I.V. Pototskaya, S.S. Shepelev, V.E. Pozherukova, Е.А. Salina, Е.S. Skolotneva, D. Hodson, M. Hovmøller, M. Patpour, A.I. Morgounov

**Affiliations:** Omsk State Agrarian University named after P.A. Stolypin, Omsk, Russia; Omsk State Agrarian University named after P.A. Stolypin, Omsk, Russia; Omsk State Agrarian University named after P.A. Stolypin, Omsk, Russia; Omsk State Agrarian University named after P.A. Stolypin, Omsk, Russia; Institute of Cytology and Genetics of Siberian Branch of the Russian Academy of Sciences, Novosibirsk, Russia; Institute of Cytology and Genetics of Siberian Branch of the Russian Academy of Sciences, Novosibirsk, Russia; CIMMYT, Addis Ababa, Ethiopia; Aarhus University, Slagelse, Denmark; Aarhus University, Slagelse, Denmark; CIMMYT-Turkey, Ankara, Turkey

**Keywords:** bread wheat, stem rust, pathotype, effective resistance genes, breeding, мягкая пшеница, стеблевая ржавчина, патотип, эффективные гены устойчивости, селекция

## Abstract

Stem rust in recent years has acquired an epiphytotic character, causing significant economic damage for wheat production in some parts of Western Siberia. On the basis of a race composition study of the stem rust populations collected in 2016–2017 in Omsk region and Altai Krai, 13 pathotypes in Omsk population and 10 in Altai population were identified. The race differentiation of stem rust using a tester set of 20 North American Sr genes differentiator lines was carried out. The genes of stem rust pathotypes of the Omsk population are avirulent only to the resistance gene Sr31, Altai isolates are avirulent not only to Sr31, but also to Sr24, and Sr30. A low frequency of virulence (10–25 %) of the Omsk population pathotypes was found for Sr11, Sr24, Sr30, and for Altai population – Sr7b, Sr9b, Sr11, SrTmp, which are ineffective in Omsk region. Field evaluations of resistance to stem rust were made in 2016–2018 in Omsk region in the varieties and spring wheat lines from three different sources. The first set included 58 lines and spring bread wheat varieties with identified Sr genes – the so-called trap nursery (ISRTN – International Stem Rust Trap Nursery). The second set included spring wheat lines from the Arsenal collection, that were previously selected according to a complex of economically valuable traits, with genes for resistance to stem rust, including genes introgressed into the common wheat genome from wild cereal species. The third set included spring bread wheat varieties created in the Omsk State Agrarian University within the framework of a shuttle breeding program, with a synthetic wheat with the Ae. tauschii genome in their pedigrees. It was established that the resistance genes Sr31, Sr40, Sr2 complex are effective against stem rust in the conditions of Western Siberia. The following sources with effective Sr genes were selected: (Benno)/6*LMPG-6 DK42, Seri 82, Cham 10, Bacanora (Sr31), RL 6087 Dyck (Sr40), Amigo (Sr24, 1RS-Am), Siouxland (Sr24, Sr31), Roughrider (Sr6, Sr36), Sisson (Sr6, Sr31, Sr36), and Fleming (Sr6, Sr24, Sr36, 1RS-Am), Pavon 76 (Sr2 complex) from the ISRTN nursery; No. 1 BC1F2 (96 × 113) × 145 × 113 (Sr2, Sr36, Sr44), No. 14а F3 (96 × 113) × 145 (Sr36, Sr44), No. 19 BC2F3 (96 × 113) × 113 (Sr2, Sr36, Sr44), and No. 20 F3 (96 × 113) × 145 (Sr2, Sr36, Sr40, Sr44) from the Arsenal collection; and the Omsk State Agrarian University varieties Element 22 (Sr31, Sr35), Lutescens 27-12, Lutescens 87-12 (Sr23, Sr36), Lutescens 70-13, and Lutescens 87-13 (Sr23, Sr31, Sr36). These sources are recommended for inclusion in the breeding process for developing stem rust resistant varieties in the region.

## Abstract (ru)

Стеблевая ржавчина пшеницы в последние годы приобрела эпифитотийный характер, нанося значительный экономический ущерб производству зерна пшеницы в отдельных областях Западной Сибири. По результатам изучения расового состава популяций стеблевой ржавчины, собранной в 2016–2017 гг. в Омской области и Алтайском крае, выявлено 13 патотипов в омской популяции и 10 – в алтайской. Диффе- ренцирование рас стеблевой ржавчины проводили с помощью тестерного набора 20 североамериканских линий-дифференциаторов Sr генов. Гены патотипов стеблевой ржавчины омской популяции авирулентны только к гену устойчивости Sr31, алтайские изоляты авирулентны, помимо Sr31, к генам Sr24, Sr30. Низкая частота вирулентности (10–25 %) патотипов омской популяции установлена для Sr11, Sr24, Sr30, а патотипов алтайской – для Sr7b, Sr9b, Sr11, SrTmp, которые неэффективны в Омской области. Полевая оценка устойчивости к стеблевой ржавчине проводилась в 2016–2018 гг. в Омской области в динамике в течение вегетационного периода у сортов и линий мягкой пшеницы из трех различных источников. Первый набор включал 58 линий и сортов яровой мягкой пшеницы с идентифицированными генами Sr, условно называемыми «питомник- ловушка» (ISRTN – international stem rust trap nursery). Второй набор включал линии яровой пшеницы из коллекции «Арсенал», отобранные ранее по комплексу хозяйственно ценных признаков и несущие пирамиду генов устойчивости к стеблевой ржавчине, в том числе интрогрессированных в геном мягкой пшеницы от дикорастущих видов злаков. Третий набор включал сорта яровой мягкой пшеницы, созданные в Омском аграрном университете по программе челночной селекции, имеющие в родословной синтетическую пшеницу с геномом Ae. tauschii. Установлено, что линии с генами Sr31, Sr40, Sr2 complex невосприимчивы к стеблевой ржавчине в условиях Западно-Сибирского региона. Выделены источники с эффективными гена- ми Sr : из питомника ISRTN – (Benno)/6*LMPG-6 DK42 (Sr31), Seri 82 (Sr31), Cham 10 (Sr31), Bacanora (Sr31), RL 6087 Dyck (Sr40), Amigo (Sr24, 1RS-Am), Siouxland (Sr24, Sr31), Roughrider (Sr6, Sr36), Sisson (Sr6, Sr31, Sr36), Fleming (Sr6, Sr24, Sr36, 1RS-Am), Pavon 76 (Sr2 complex); из коллекции «Арсенал» – № 1 BC1F2 (96 × 113) × 145 × 113 (Sr2, Sr36, Sr44), № 14а F3 (96 × 113) × 145 (Sr36, Sr44), № 19 BC2F3 (96 × 113) × 113 (Sr2, Sr36, Sr44), № 20 F3 (96 × 113) × 145 (Sr2, Sr36, Sr40, Sr44); сорта Омского аграрного университета – Элемент 22 (Sr31, Sr35), Лютесценс 27-12, Лютес- ценс 87-12 (Sr23, Sr36), Лютесценс 70-13, Лютесценс 87- 13 (Sr23, Sr31, Sr36). Выделенные источники рекомен- дуются для включения в селекционный процесс при создании сортов, устойчивых к стеблевой ржавчине в условиях региона.

## Introduction

Stem rust of wheat caused by Puccinia graminis f. sp. tritici Erikss. for a long time had a weak manifestation in the territory of Western Siberia and only in the recent years acquired an epiphytotic nature, causing significant economic damage for wheat production in the region. First of all, this is due to the deterioration of the phytosanitary situation in the region, the general trend of climate warming and cultivation of susceptible wheat varieties on large area (Shamanin et al., 2015, 2016a). The threat of stem rust race Ug99 appearance and the emergence of new pathotypes of this race, affecting varieties with genes Sr24 and Sr36 present a serious threat for wheat production in West Siberian region. Genetic diversity of cultivated wheat varieties for resistance to Ug99 and stem rust in general is very limited (Shamanin et al., 2016b).

Enhancement of genetic resistance to pathogens can be solved germplasm exchange, and also cultivation of varieties with different level of resistance to diseases and to different races. Crop protection is necessary to restrain the evolution of pathogens and the emergence of new virulent races. Such programs are widely used in Europe and America. The duration of the variety cultivation in advanced countries is 3–4 years, while in Russia – 7–10 years (Sanin, 2016). In this regard, the breeding of spring wheat varieties, which have a diverse genetic basis of resistance to stem rust, is very relevant.

Since the 1950s, many resistance genes introduced into bread wheat have lost their effectiveness (Singh et al., 2008). The most significant genes for breeding practice are Sr2, Sr23, Sr24, Sr25, Sr31, Sr33, Sr36, Sr38, Sr45, Sr50, SrTmp, Sr1RS^Amigo^ (Singh et al., 2015).

Introgression of resistance genes of wild and cultivated wheat relatives allows to expand the genetic diversity of varieties and contributes to their long-term protection (Leonova et al., 2014). To date, about 86 Sr genes have been identified, of which 26 stem rust resistance genes have been transferred into bread wheat from other cereal species (McIntosh et al., 2013). For example, T. turgidum was the source of the stem rust resistance genes Sr2, Sr9d, Sr9e, Sr9g, Sr11, Sr12, Sr13, Sr14, and Sr17, of which the Sr2, Sr13, and Sr14 genes are effective against Ug99 race; T. monococcum was the source of Sr21, Sr22, and Sr35 genes (Singh et al., 2011).

Genes that caused the resistance to stem rust have been introduced into wheat gene pool from the genome of various Aegilops L. species: Ae. speltoides – Sr32, Sr39, Sr47; Ae. comosa – Sr34; Ae. ventricosa – Sr38 (Schneider et al., 2008). Ae. tauschii contributed genes Sr33, Sr45, Sr46 (Kerber, Dyck, 1979). Direct hybridization of T. aestivum with Ae. tauschii and following backcrosses allowed introduction of new resistance genes SrTA1662, SrTA1017, and SrTA10187 effective against Ug99 race (Olson et al., 2013). The search of new resistance genes in wild wheat relatives continues, for example, G. Yu et al. (2017) identified two new Sr genes in Ae. sharonesis.

One of the objectives of Kazakh-Siberian Spring Wheat Improvement Network (KASIB) is expanding of the genetic polymorphism of new varieties, including resistance to harmful diseases (Gomez-Becerra et al., 2006). This is based on shuttle breeding with CIMMYT (Mexico). Varieties and breeding lines developed through shuttle breeding with participation of Ae. tauschii and T. dicoccum, as well as lines of the “Arsenal” collection, which have wild species in their pedigree are of interest for breeding for resistance to stem rust in the region.

The aim of the research was analysis of the racial composition of the Western-Siberian stem rust population, resistance assessment of spring bread wheat lines and varieties with identified resistance genes and identification of the sources with effective Sr genes for breeding under Western Siberian conditions.

## Material and methods

The racial composition of Puccinia graminis f. sp. tritici populations collected in 2016–2017 in Omsk region (15 entries of the nursery KASIB-16, Omsk State Agrarian University (SAU)) and Altai region (12 breeding samples, Altay Breeding Center) were analyzed in the Global Rust Reference Center (GRRC, Denmark; http://agro.au.dk/forskning/internationaleplatforme/wheatrust).

Selection of single pustule isolates according to requirements of GRRC protocols (www.wheatrust.org) was carried out. Monopustule isolates were reproducted to identify race Ug99 with usage of the test PCR-Stage 1. A total of 19 single pustule isolates were selected from Omsk population and 20 – from Altai population (Table 1).

**Table 1.**
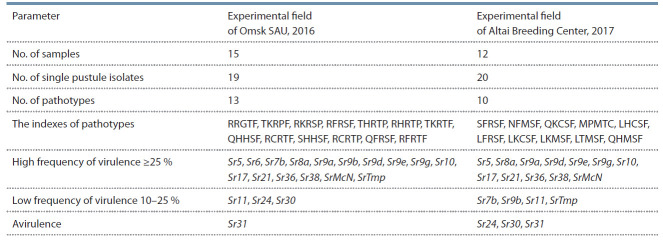
Phenotypic composition and virulence of pathotypes of Puccinia graminis f. sp. tritici in Omsk and Altai regions (2016–2017)

Differentiation of stem rust races was performed with use of the set of 20 North American differentiator lines containing Sr genes: Sr5 (ISr5-Ra), Sr21 (CnS_Triticum monoc. Deriv.), Sr9e (Vernstein), Sr7b (ISr7b-Ra), Sr11 (ISr11- Ra), Sr6 (ISr6a-Ra), Sr8a (ISr8a-Ra), Sr9g (CnSr9g), Sr36 (W2691SrTt-1), Sr9b (W2691Sr9b), Sr30 (BtSr30Wst), Sr17+13 (Combination VII), Sr9a (ISr9a-Ra), Sr9d (ISr9d- Ra), Sr10 (W2691Sr10), SrTmp (CnsSrTmp), Sr24 (LcSr24Ag), Sr31 (Benno Sr31/6*LMPG), Sr38 (VPM-1), SrMcN (McNair 701). Infected plants were evaluated in 14–16 days after inoculation according to modified E.C. Stakman scale (Roelfs, Martens, 1988). Virulence phenotypes were classified according to North American system (Jin et al., 2008).

The varieties and lines of bread wheat from three germplasm sets were evaluated in Omsk at least 4–5 times for reaction to stem rust on scales recommended by Koyshibaev et al. (2014). The type of reaction on E.B. Mains and H.S. Jackson scale (1926) and severity – on modified Peterson scale (Peterson et al.,1948) were considered: 0 – immunity, uredopustules not formed; R (Resistance – high resistance), 1 score, severity 5–10 %; MR (Moderately resistant – average resistance), 2 score, severity 10–25 %; M (heterogeneous type), pustules of different sizes, surrounded by chlorotic and necrotic spots or without them; MS (Moderately susceptible – average susceptibility), 3 score, severity 40–50 %; S (Susceptible – susceptibility), 4 score, severity more than 60 %.

In 2016–2018, International Stem Rust Trap Nursery with 58 genotypes with identified Sr genes was evaluated to Omsk stem rust population (Table 2). Varieties and lines of nurserytrap were sown manually in 100 cm-long rows with stem rust resistant (Element 22) and susceptible checks (Chernyava 13) alternating every entries.

**Table 2.**
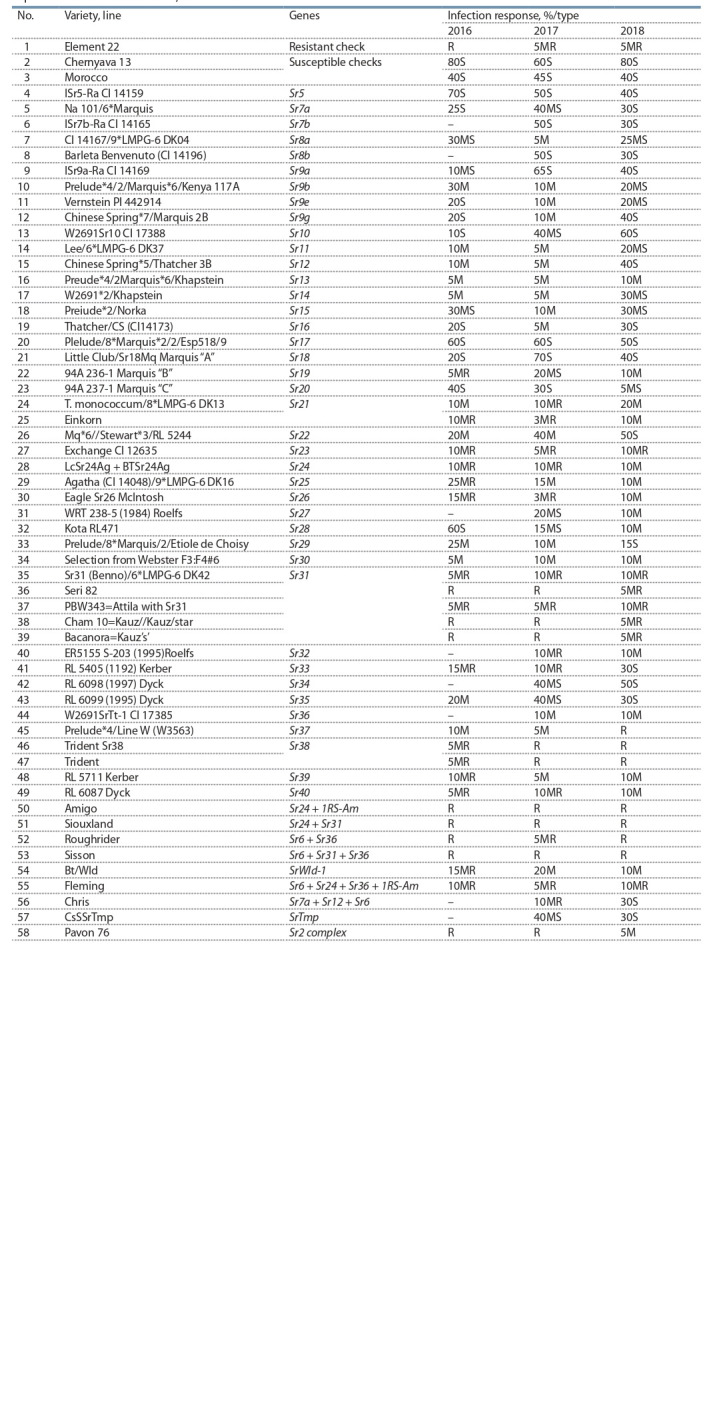
Results of evaluation of lines and varieties with identified Sr genes on resistance/susceptibility to stem rust, experimental field of Omsk SAU, 2016–2018

In 2015, 9 spring wheat lines originating from wide crosses “Arsenal” collection were kindly provided by I.F. Lapochkina for evaluation in Omsk. These lines carry a pyramid of stem rust resistance genes (Lapochkina et al., 2017) – No. 1 [BC_1_F_2_ (96 × 113) × 145 × 113]; No. 13, 14а [F_3_ (96 × 113) × 145]; No. 16, 17, 17а [BC_1_F_4_ (96 × 113) × 113]; No. 19 [BC_2_F_3_ (96 × 113) × 113]; No. 20, 22а [F_3_ (96 × 113) × 145]. The lines were studied in 2016–2018 in un-replicated trial with the plot size of 2 m^2^.

Nine spring wheat varieties and breeding lines from advanced yield trial at Omsk SAU developed through utilization of synthetic wheat with the Ae. tauschii genome (Lutescens 24-12 (Kasibovskaya), Lutescens 27-12, Lutescens 87- 12, Lutescens 70-13, Lutescens 87-13, Lutescens 88-13 (Silantiy), Lutescens 124-13, Lutescens 53-15, Lutescens 128- 15) were evaluated for stem rust resistance and other traits in 2016–2018. The plot size was 25 m2 with four replications. The checks were Pamyati Azieva (early maturing), Duet (medium maturing), and Element 22 (late maturing).

Sr genes of Omsk SAU varieties were identified using molecular markers: Xsts638 – Sr15, Xcfa2123 – Sr22, Xgwm210 – Sr23, Xscs73 – Sr24, Xwmc221 – Sr25, BE518379 – Sr26, Xscm09 – Sr31, SCS421 – Sr34, Xcfa2170 – Sr35, Xstm773-2 – Sr36, Ventriup-LN2 – Sr38, Lr34plus – Sr57, according to established protocol (http://maswheat.ucdavis.edu/protocols/StemRust/index.htm). The resistance genes of spring bread wheat lines and varieties from nursery-trap and from collection “Arsenal” were identified earlier (McIntosh et al., 2013, 2017; Lapochkina et al., 2017).

In 2016, weather conditions in Omsk region were relatively dry, which contributed to moderate development of stem rust. In 2017, there was an intensive development of the disease, the degree of severity of susceptible accessions varied within 20S–80S. In 2018 high severity of stem rust was observed as the growing season was characterized by cool weather and more precipitation. The degree of severity of susceptible accessions was 30S–80S.

## Results

The race composition analysis of stem rust populations identified a significant number of pathotypes: in the Omsk population – 13 and in Altai population – 10 (see Table 1). Unlike many regions of the world where stem rust is a harmful disease for decades, for example in Krasnodar region of Russia (Ablova et al., 2016), for Western Siberia this is surprising result considering a short period of time since its appearance. Most of the identified pathotypes of stem rust population in Omsk and Altai regions were not identical in virulence to the pathotypes, which were found in recent years in Asia and Africa (http://wheatrust.org/fileadmin/www). In all studied Western-Siberian populations of P. graminis Ug99 and Sicilian races were not identified. Genes of stem rust pathotypes of Omsk population were avirulent only to Sr31 gene, while Altai pathotypes were avirulent to Sr31, Sr24, and Sr30.

Low frequency of virulence (10–25 %) of Omsk population pathotypes was established for Sr11, Sr24, Sr30 genes, for Altai population – for Sr7b, Sr9b, Sr11, SrTmp genes, which were ineffective in Omsk region. The results of laboratory evaluation of virulence of P. graminis pathotypes collected in Omsk region were confirmed by field of trap nursery with identified Sr genes (see Table 2).

Genotypes with Sr31: Sr31(Benno)/6*LMPG-6 DK42, Seri 82, PBW343=Attila with Sr31, Cham 10=Kauz//Kauz/ star, Bacanora=Kauz’s’ showed high level of resistance to Omsk stem rust population in all years of study (2016–2018). Line 28 LcSr24Ag + BTSr24Ag with Sr24 gene was characterized by moderate resistance. For some Sr genes, resistant type of reaction under epiphytotic conditions was observed on the stage of adult plants, and susceptible type – on the seedling stage in the laboratory conditions.

For example, variety Trident (entries 46 and 47) with Sr38 gene had high resistance (R–5MR) in the field; variety Einkorn (entry 25) with Sr21 gene, and line W2691SrTt-1 CI 17385 (entry 44) with Sr36 gene had moderate resistance (10M) in the field conditions. In the laboratory conditions the seedlings plants with above mentioned genes were classified as susceptible. Genotypes of ISRTN nursery with a gene pyramid had high resistance to stem rust in all years of research: entry 50 Amigo (Sr24 + 1RS-Am), entry 51 Siouxland (Sr24 + Sr31), entry 52 Roughrider (Sr6 + Sr36), entry 53 Sisson (Sr6 + Sr31 + Sr36), entry 55 Fleming (Sr6 + Sr24 + Sr36 + 1RS-Am). The results of stem rust resistance evaluation of “Arsenal” collection and Omsk SAU germplasm are presented in Table 3.

**Table 3.**
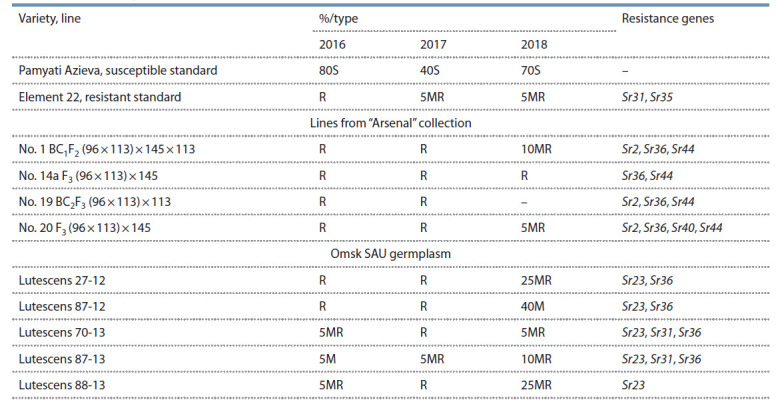
Results of evaluation of lines and varieties with identified Sr genes on resistance/susceptibility to stem rust, experimental field of Omsk SAU, 2016–2018

Lines from “Arsenal” collection are of great interest as sources of resistance to pathogen since they possess the gene pyramid: Sr2 (T. turgidum), Sr36, Sr40 (T. timopheevii), Sr44 (Th. intermediate). The pedigree of selected lines contains spring wheat line 13/00/i-4 with 7 resistance genes: Sr2, Sr36, Sr39, Sr40, Sr44, Sr47, Sr15, and winter line GT 96/90 with genes Sr15, Sr24, Sr31, Sr36, Sr40, Sr47 (Lapochkina et al., 2017).

In Omsk SAU varieties 3 resistance genes were identified: Sr23, Sr31, Sr36. Variety Element 22, which has winter wheat Aurora in its pedigree also possesses wheat-rye translocation 1BL.1RS with Sr31 gene (Shamanin et al., 2016b). The combination of effective resistance genes Sr31 and Sr35 in this variety results a high level of resistance to stem rust. Element 22 is one of the few varieties with combined resistance to stem and leaf rust. It was included into State register of breeding achievements in Western Siberian region. This variety is the check of the late maturity group at the State Variety Trials in Omsk region.

Stem rust resistant breeding lines Lutescens 27-12, Lutescens 70-13, Lutescens 87-13, Lutescens 88-13 were selected from a cross Lutescens 30-94*2/3/T. dicoccon PI 94625/ Ae. squarrosa (372)//3*Pastor involving Kazakhstan spring wheat line Lutescens 30-94 and CIMMYT line developed by hybridization of synthetic wheat with variety Pastor. The line Lutescens 87-12 originated from a cross Kazakhstanskaya 25/2*Attila/3/T. dicoccon PI 94625/Ae. squarrosa also involving synthetic wheat. Omsk SAU germplasm possessed different combinations of genes Sr23, Sr31, and Sr36.

## Discussion

In modern conditions, stem rust is the most dangerous disease for grain production in Western Siberia. In the epiphytotic years the grain losses of wheat in the region were about 2 million tons. Unfortunately, stem rust resistant varieties included into the State register occupy about 10–15 % of the total wheat sowing area in the region. In 2015–2016, evaluation of spring wheat varieties at Moskalenskiy State Variety Trial of Omsk region (southern forest-steppe zone) demonstrated that out of 57 varieties tested only Element 22 (Sr31 + Sr35), Omskaya 37, Sigma, Uralosibirskaya (Sr31), and Sigma 2 (Sr31 + Sr25) were resistant to stem rust (5–15MR). The other varieties were affected by pathogen in medium and high degree requiring the use of chemical protection (Lapochkina et al., 2017). Previously, Shamanin et al. (2016b) identified the stem rust resistance genes in the germplasm developed by breeding institutions of Western Siberia. High frequency of genes Sr25, Sr31, and their combination was observed. High variability of the race composition of the pathogen population, as shown in our studies, and the uniformity of resistance genes to stem rust in cultivated varieties, threaten grain production stability in Western Siberia.

The breeding strategy should focus on limiting disease development in the region. The study of the populations of P. graminis, formed on wheat in the different regions, is very essential to guide the breeding efforts. There were no clones avirulent to Sr24 gene in Omsk population of P. graminis while in Altai region there were no clones virulent to Sr24, which remains its effectiveness in Novosibirsk region (Skolotneva et al., 2018). The results of the population composition comparison suggest that Omsk and Altai subpopulations have relatively independent sources of genetic diversity and the contact zone. Western Siberian population of P. graminis has quite complex structure. Two subpopulations are assumed to exist: Omsk and Altai – with independent sources of genetic diversity, and zone of genotypic exchange on wheat crop in Novosibirsk region (Skolotneva et al., 2020).

Omsk stem rust population analysis showed that the spectrum of effective resistance genes has narrowed due to losses of some genes to the local population of P. graminis.

Highly resistant varieties and lines of ISRTN nursery were identified: Sr31 (Benno)/6*LMPG-6 DK42, Seri 82, Cham 10, Bacanora (Sr31), RL 6087 Dyck (Sr40), Amigo (Sr24, 1RS-Am), Siouxland (Sr24, Sr31), Roughrider (Sr6, Sr36), Sisson (Sr6, Sr31, Sr36), Fleming (Sr6, Sr24, Sr36, 1RS-Am), Pavon 76 (Sr2 complex). Selected varieties and lines are recommended for using as sources of resistance in breeding programs to create resistant wheat varieties to stem rust. Effective resistance genes Sr31, Sr40, Sr2 complex, and their combinations with ineffective genes are recommended for use in breeding, taking into account the constant rotation, combination of genes of nonspecific resistance, as well as the possibility of infection threat from neighboring territory.

The resistance gene Sr2, widely used in breeding for resistance to virulent stem rust races, is common in commercial varieties in a number of countries around the world, particularly in the United States, Australia, India, and Mexico. This gene is practically absent in the commercial varieties of Russian Federation, however, for effective protection against stem rust, its pyramiding with other resistance genes is recommended (Baranova et al., 2015).

For the development of varieties with long-term resistance, the strategy of combining genes responsible for different types of resistance in one genotype is used. Pyramiding of specific resistance genes (Sr11, Sr24, Sr30, and Sr31) with APR gene Sr2, which causes the slow development of the disease (slow rusting), will provide longer protection of wheat crops from stem rust in Western Siberia in the present phytosanitary situation.

In this regard, the lines from “Arsenal” collection – No. 1 BC_1_F_2_ (96 × 113) × 145 × 113 (Sr2, Sr36, Sr44); No. 14а F_3_ (96 × 113) × 145 (Sr36, Sr44); No. 19 BC_2_F_3_ (96 × 113) × 113 (Sr2, Sr36, Sr44); No. 20 F_3_ (96 × 113) × 145 (Sr2, Sr36, Sr40, Sr44) represent a promising starting material for breeding and creation of varieties with long-term resistance.

It is justified to include resistance sources to stem rust with minimum number of negative traits that reduce their breeding value. In this regard, stem rust resistant germplasm from Omsk SAU with identified effective genes Element 22 (Sr31, Sr35), Lutescence 27-12, Lutescence 87-12 (Sr23, Sr36 ), Lutescence 70-13, Lutescence 87-13 (Sr23, Sr31, Sr36 ), Lutescence 88-13 (Sr23) are valuable starting material for breeding in the region.

## Conclusion

Thus, the genetic similarity of spring wheat varieties on stem rust resistance genes cultivated over large areas in Western Siberia, and the predominance of varieties with race specific resistance genes contribute to spreading and high variability of the pathogen. The lines from collection “Arsenal” – No. 1 BC_1_F_2_ (96 × 113) × 145 × 113 (Sr2, Sr36, Sr44), No. 14а F_3_ (96 × 113) × 145 (Sr36, Sr44), No. 19 BC_2_F_3_ (96 × 113) × 113 (Sr2, Sr36, Sr44), No. 20 F_3_ (96 × 113) × 145 (Sr2, Sr36, Sr40, Sr44), varieties of Omsk Agrarian University – Element 22 (Sr31, Sr35), Lutescens 27-12, Lutescens 87-12 (Sr23, Sr36), Lutescens 70-13, Lutescens 87-13 (Sr23, Sr31, Sr36) are recommended for inclusion into breeding process of the creation of resistant to stem rust varieties in the region. Further monitoring of the virulence of stem rust pathogen and coordination strategy of breeding programs in Western Siberia, and neighboring regions of the Kazakhstan Republic is recommended. Incorporation of effective resistance genes, in particular Sr2 and Sr40, will improve the phytosanitary situation and expand the segment of resistant varieties in the region.

## Conflict of interest

The authors declare no conflict of interest.
